# Investigation of short-term surgical complications in a low-resource, high-volume dog sterilisation clinic in India

**DOI:** 10.1186/s12917-018-1378-3

**Published:** 2018-02-27

**Authors:** I. Airikkala-Otter, L. Gamble, S. Mazeri, I. G. Handel, B. M. de C. Bronsvoort, R. J. Mellanby, N. V. Meunier

**Affiliations:** 1 Worldwide Veterinary Service, 4 Castle Street, Cranborne, Dorset, BH21 5PZ UK; 2 The Epidemiology, Economics and Risk Assessment (EERA) Group, The Roslin Institute and the Royal (Dick) School of Veterinary Studies (R(D)SVS), Easter Bush, Midlothian, EH25 9RG UK; 3 The Royal (Dick) School of Veterinary Studies (R(D)SVS) and the Roslin Institute, Hospital for Small Animals, Easter Bush Veterinary Centre, Midlothian, EH25 9RG UK

**Keywords:** Sterilisation surgery, Ovariohysterectomy, Castration, Surgical complications, Companion animal, Welfare

## Abstract

**Background:**

Surgical sterilisation is currently the method of choice for controlling free-roaming dog populations. However, there are significant logistical challenges to neutering large numbers of dogs in low-resource clinics. The aim of this study was to investigate the incidence of short-term surgical complications in a low-resource sterilisation clinic which did not routinely administer post-operative antibiotics.

The medical records of all sterilisation surgeries performed in 2015 at the Worldwide Veterinary Service International Training Centre in Tamil Nadu, India were reviewed (group A) to assess immediate surgical complications. All animals in this group were monitored for at least 24 h post-surgery but were not released until assessed by a veterinarian as having uncomplicated wound healing. In the second part of this study from August to December 2015, 200 free-roaming dogs undergoing sterilisation surgery, were monitored for a minimum of 4-days post-surgery to further assess postoperative complications (group B).

**Results:**

Surgery related complications were seen in 5.4% (95%CI, 4.5–6.5%) of the 1998 group A dogs monitored for at least 24 h, and in 7.0% (3.9–11.5%) of the 200 group B dogs monitored for 4 days. Major complications were classed as those requiring an intervention and resulted in increased morbidity or mortality. Major complications were seen in 2.8% (2.1–3.6%) and 1.5% (3.1–4.3%) of group A and B, respectively. Minor complications requiring little or no intervention were recorded for 2.6% (1.9–3.4%) for group A and 5.5% (2.8–9.6%) for group B. There was no evidence for a difference in complication rates between the two groups in a multivariate regression model.

**Conclusion:**

This study demonstrated that high volume, low-resource sterilisation of dogs can be performed with a low incidence of surgical complications and low mortality.

## Background

Developing strategies to effectively control the size and impact of free-roaming dog populations is an important public health priority in many countries. Free-roaming dogs are susceptible to a number of welfare concerns such as malnutrition, disease, and traffic accidents [1]. In addition, large free-roaming dog populations can lead to conflicts with humans. These conflicts include zoonotic diseases such as rabies, nuisance through noise and pollution, aggressive behaviour towards people, especially children, and predation of livestock and wildlife [2, 3]. Implementing dog population control measures has been shown to reduce dog bites [4], lower the vaccination threshold required for effective rabies control [5], as well as increase body condition scores and decrease the prevalence of numerous diseases in the dog population [6].

A wide range of dog population control techniques have been reported. These include large-scale culling operations, which have now been shown to be ineffective because immigration and increased birth rates quickly compensate for the losses [7]. Additionally, culling dogs is not an effective approach to reduce the impact of zoonotic diseases such as rabies [7, 8]. Chemical castration has been used as an alternative to surgery [9] and immunocontraceptives are under development [10, 11]. Despite these advances, surgical sterilisation clinics are currently the main control method advocated to control free-roaming dog populations [12]. However, undertaking surgical sterilisation on a large scale is logistically challenging in many parts of the world, notably in low income settings where the need to control these populations is often the greatest. In addition, surgical sterilisation can be associated with major complications including haemorrhage, ovarian remnant syndrome, stump pyometra, adhesions, and wound dehiscence or infection, as well as anaesthetic complications and drug reactions [13, 14]. Moreover, there are few reports which describe the incidence of surgical complications in clinics which sterilise large numbers of dogs in low income settings.

The objective of this study was to investigate the incidence of surgical complications in dogs in a low-resource sterilisation clinic which did not administer routine postoperative antibiotics. This was done by assessing peri- and post-operative complications following sterilisation surgery, including deaths, iatrogenic surgical complications and wound breakdown in a cohort of 200 dogs over a period of 4 days. In addition, surgery records were assessed for all dogs in the same year for a minimum 24 h follow-up period.

## Methods

### Study site

The Worldwide Veterinary Service (WVS) International Training Centre (ITC) in India has been performing male and female dog sterilisation surgeries since 2010 to control the free-roaming dog population in the Nilgiris district of Tamil Nadu. The sterilisation programmes are conducted according to the Animal Birth Control (ABC) guidelines, as recommended by the Animal Welfare Board of India [15]. During 2010–2015, approximately 14,000 dogs were neutered in the training centre. The WVS ITC presented a regular two-week training course teaching high-standard sterilisation techniques to veterinarians and international veterinary student participants. All surgeries were conducted under the supervision of ITC veterinary instructors. Free-roaming dogs from the nearby towns and villages were caught and brought in to the clinic by the WVS ITC team of trained animal handlers. The average number of surgeries undertaken at the WVS ITC clinic was 20 surgeries per working day during the study period.

Ethics approval for this study was attained from the University of Edinburgh, Veterinary Ethics Research Committee (VERC 114.16). The neutering programme was approved by the Animal Welfare Board of India.

### Study design

For the first part of this study, all surgical records at the WVS ITC from January to December 2015 were reviewed (group A). All dog sterilisation case record sheets were entered into a database using a purpose-designed form on a smartphone based application (The Rabies App, Mission Rabies; WVS Data Collection App, WVS; 2016). Records were excluded if details of surgical monitoring and recovery were missing.

In the second part of the study, 200 free-roaming dogs were monitored for complications after surgery for a minimum of 4 days between August and December 2015 (group B). Kennel space was a major limitation and dogs were enrolled as space became available. This resulted in batches of all dogs operated on certain days being included in the study as a kennel became free, with no dogs enrolled on other days even though surgery took place.

### Surgical protocol

Premedication of xylazine (2 mg/kg IM) and butorphanol (0.1 mg/kg IM) was administered in the kennel based on an estimated weight. Once sedated, dogs were weighed for accurate medication dosages and an intravenous catheter was placed in all cases. Intravenous fluids (0.9% normal saline) were administered at 10 ml/kg/h throughout the surgery. Dogs were induced with propofol (1 mg/kg IV) and diazepam (0.25 mg/kg IV). After induction, all dogs received amoxicillin-cloxacillin (20 mg/kg IV), meloxicam (0.2 mg/kg IV), tramadol (4 mg/kg IV) and ivermectin (200μg/kg SC). Lignocaine was given as constant rate infusion for analgesia (1.2 mg/kg/h) and male dogs were injected subcutaneously with lignocaine as a prescrotal local block (20 mg SC). Maintenance of the anaesthesia was achieved with a propofol bolus (1 mg/kg IV) every 6–10 min to effect.

After induction dogs were prepared for surgery by manually shaving the surgical site and cleaning the surgical field with a chlorhexidine surgical scrub solution for 5 min. The preparation of the surgical field was completed with a final spray of isopropyl alcohol. Sterile drapes and surgical instruments were used for each patient. Surgical caps, masks and sterile gloves were worn by all surgeons. Standard aseptic technique was followed in all surgical procedures, including surgical scrubbing of hands with an iodine-based scrub solution and aseptic handling of instruments and consumables after autoclaving. Surgeons replaced their gloves if a break in aseptic technique occurred.

Animals were examined before surgery for pregnancy or cryptorchidism. Ovariohysterectomy was performed with a ventral midline incision according to standard techniques [16]. Bilateral orchidectomy was done with a midline prescrotal incision. Catgut was used for internal ligatures and absorbable suture material for closing the muscle, subcutaneous and intradermal layers in a continuous pattern. The majority of dogs were operated on by course participants (students or veterinary surgeons with limited experience) under the direct supervision of a surgically scrubbed veterinary instructor. A small number of animals were sterilised by experienced surgeons for demonstration purposes. The surgical protocol followed was the same for all dogs operated at the WVS ITC.

All ownerless dogs were kept overnight after surgery and received meloxicam (0.2 mg/kg SC) and a rabies vaccine on the following day. Meloxicam injections were repeated daily for dogs remaining on site. Post-operative antibiotics were not given unless clinically indicated such as in patients with visible signs of infection or systemic illness. All free-roaming dogs were monitored for at least 24 h post-surgery before being released.

### Post-operative assessment

Dogs were monitored daily by WVS ITC veterinary instructors until release. Wound scores were recorded each morning on a scale 0–4 (Table 1, with 0 = perfect healing, and 4 = open wound) and any interventions or complications were recorded. Pain scores were additionally recorded dependant on the visual appearance of the dog and its reaction to gentle touch around the surgical wound, scored with a pain scale from ‘0’ no pain, to ‘9’ excruciating pain, based on the scale by Mathews, (2000) [17] (Table 2).

**Table 1:** Post-operative wound scoring scale used at the WVS ITC

Wound score	Wound description
0	Perfectly healing wound, edges in apposition
1	Mild redness on the skin around the wound
2	Swelling or discharge or exposed subcutis
3	Partial opening of the wound
4	Complete opening of the wound

**Table 2:** Post-operative pain scoring scale used at the WVS ITC

Pain score	Clinical signs often associated with degree of pain
0 No pain	Bright, eating, sleeping comfortably, grooming, affectionate
1 Mild discomfort	Eats, sleeps, resists surgical palpation, not depressed
2 Mild pain	Picks at food, guards surgical area, slightly depressed
3 Mild to moderate	Inappetant, guards/ looks/ licks/ chews surgical area, unrelaxed, whimpers
4 Moderate	Depressed, reluctant to move, aggressive, may vocalise, mydriasis
5 Increased moderate	As 4, but more pronounced
6 Moderate to severe	Very depressed, will not move even to urinate, vocalises often
7 Severe	Motionless, extremely depressed, vocalises
8 Increased severe	As 7, hyperalgesic wherever touched, trembling
9 Excruciating	Piercing screams, nearly comatose

Free-roaming dogs were returned to their original location 1–5 days after surgery, depending on the healing of the wound as well as logistics and schedules of the release and catching vehicles. Dogs with a wound healing score of 0 or 1 would be marked for release. Dogs with a wound score of 2 or above, or any additional complications, were kept for monitoring or treatment as required. All dogs in group B were kept for a minimum of four days regardless of wound score.

### Analysis

Complications were entered into the database as free text comments from the surgical monitoring and follow-up sheets. Any surgically related complication which developed during or after surgery in the follow-up period was categorised into a) major complications, requiring intervention or resulting in high morbidity or death; b) minor complications, requiring observation; and c) surgical site specific, relating to the incision wound. These categories were not mutually exclusive.

The excluded patient population, those with incomplete patient records, were compared to the study group with univariate analysis of factors with Pearson’s Chi-square test, Fisher’s exact test, or Students T-test, as appropriate. A logistic regression model was used to look at the association of study group, age, sex, weight, and surgery time (minutes), with an interaction term for sex and surgery time, on the three categories of complications versus uncomplicated surgery. Age was classified into three groups for the purpose of analysis: < 1 year, 1–2 years, > 3 years. All analysis was conducted in R Statistical Software [18].

## Results

### Total records

The records of 2395 surgically sterilised dogs were examined for 2015. Of these, 197 records were excluded due to incomplete information on wounds scores or surgical monitoring. There was no difference between those included or excluded with regard to: sex, age, surgery time, major, incidental or surgery site complications. Minor complications were lower in the excluded group (*p* = 0.04). Of those excluded from the study, 2 dogs were euthanised during surgery for pre-existing health conditions with poor prognosis.

Paper records were available for 2198 free-roaming dogs which were included in the study. Of these records, 1998 dogs were potentially monitored during and after surgery for at least 24 h until fit for release and 200 dogs were monitored for the minimum period of four days.

### Group A 24-h monitoring

The short-term monitoring study comprised of 1998 surgical records of dogs sterilised at the WVS ITC clinic in 2015. There were 932 ovariohysterectomies and 1066 castrations, including 44 pregnant bitches and 12 cryptorchid dogs. The median age was 2 years (range 0.2–12.5). Mean surgical times were 95.7 min for ovariohysterectomy (SD 25.3) and 50.2 min for castration (SD 18.5). Based on assessment of the surgical wound on the first day following surgery, 70.2% of dogs were released, and subsequently, 91.2% of dogs had been released within two days of surgery.

In total, 108 dogs (5.4%; 95%CI 4.5–6.5%) had at least one surgery related complication in this group (Table 3). The major complications were seen in 56 dogs (2.8%; 2.1–3.6%). Two anaesthetic deaths were reported, and one dog died two days after surgery with haemorrhagic gastroenteritis. Dehiscence of the wound resulted in a scrotal ablation in one case. A further three scrotal ablations were performed at the time of the initial surgery, but the reasons for these were not clearly documented. Surgical intervention was required in three dogs intra-operatively with major haemorrhage, two specifically with torn ovarian ligaments, and one female dog re-operated immediately following ovariohysterectomy due to suspected internal bleeding; all recovered without further incident. Anaesthetic reasons for intervention included tremors or seizures requiring diazepam (*n* = 10). Further interventions including wound flushing (*n* = 17) and further antibiotic administration (*n* = 4). Where an alternative reason was not given for post-operative antimicrobial use, these were considered related to the surgical wound (*n* = 6). Pyometra was diagnosed at surgery and treated in 11 dogs. Minor complications were seen in 52 dogs (2.6%; 1.9–3.4%). These included mild wound swelling or discharge, notable blood loss during surgery, the development of diarrhoea, and postoperative hypothermia (Table 4). Complications specifically related to the surgical site were seen in 54 of 1996 dogs (2.7%; 2.0–3.5%).

**Table 3:** Complication frequency of dogs undergoing sterilisation surgery in 2015

	Group A – 24 h monitoring			Group B – 4 day monitoring		
Complications	n (N = 1998)	Percent	95% CI	n (N = 200)	Percent	95% CI
Surgical (Any)	108	5.4	4.5–6.5	14	7.0	3.9–11.5
Major surgical (required intervention)	56	2.8	2.1–3.6	3	1.5	3.1–4.3
Minor surgical (observation only)	52	2.6	1.9–3.4	11	5.5	2.8–9.6
Surgical site related (post-operative)	54^a^	2.7	2.0–3.5	9	4.5	2.1–8.4

(^a^N = 1996)

**Table 4:** Reported complications associated with surgery presented in major and minor subclasses

	Group A 24 h (N = 1998)		Group B 4-day (N = 200)	
Complication	Number	Percent	Number	Percent
a) Surgery related complications				
Major complications				
Postoperative mortality	1	(0.1)	0	(0.0)
Anaesthetic death	2	(0.1)	0	(0.0)
Scrotal ablation required	4	(0.2)	1	(0.5)
Surgical intervention	4	(0.2)	0	(0.0)
Anaesthetic intervention	10	(0.5)	0	(0.0)
Antibiotic treatment	10	(0.5)	0	(0.0)
Pyometra	11	(0.6)	0	(0.0)
Surgical wound flushing	17	(0.9)	2	(1.0)
Minor complications				
Hypothermia	4	(0.2)	1	(0.5)
Incisional discharge	5	(0.3)	3	(1.5)
Incision wound swelling	6	(0.3)	0	(0.0)
Moderate blood loss	7	(0.4)	0	(0.0)
Moderate scrotal swelling	8	(0.4)	3	(1.5)
Diarrhoea	8	(0.4)	3	(1.5)
Cardiac rhythm abnormalities	8	(0.4)	0	(0.0)
Procedure related	10	(0.5)	1	(0.5)

This case number was supported by 92.8% of dogs having a wound healing score of ‘0–1’ (good healing to mild redness) on the first day post-op, 7.1% with a score of ‘2’ (swelling or discharge), and only one animal with a score of ‘3’ (partial opening). The pain score was ‘0’ (no pain) in 72.3% of dogs, ‘1’ (mild discomfort) in 25.0%, and ‘2–3’ (mild to moderate) in 2.7% of dogs.

### Group B 4-day monitoring

The 4-day follow-up study evaluated the immediate surgical complications and short-term wound healing of 200 dogs with complete monitoring records (86 females and 114 males) operated on between August and December 2015. Two females were pregnant and one male was a cryptorchid. The median age was 2 years (range 0.3–12.0 years). The mean surgical times were 92.2 (SD 22.5) and 52.2 (SD 19.8) minutes for the ovariohysterectomy and castrations, respectively.

Fourteen of the 200 dogs (7.0%, 95%CI 3.9–11.5%) had at least one surgery related complication (Table 3). Three major complications, 1.5% (3.1–4.3%), were seen in the prospective group. These included one scrotal ablation which was performed in a dog at the time of the initial surgery due to a scrotal incision made during shaving, and two further cases required flushing of the surgical wound. Minor complications were seen in 11 of 200 dogs (5.5%; 2.8–9.6%), including moderate scrotal swelling (*n* = 3), wound discharge (*n* = 3), diarrhoea (*n* = 3), one shaving inflicted wound, and postoperative hypothermia (*n* = 1). Nine cases were specifically related to the surgical site (4.5%, 2.1–8.4%). No wound breakdown or post-operative wound infection was observed. These results can be seen in Table 4.

**Fig. 1:**
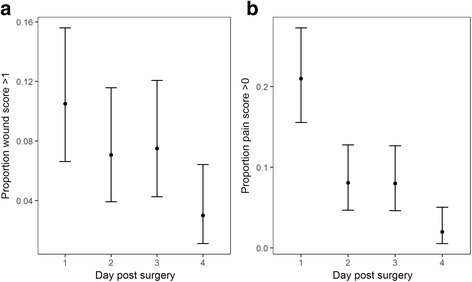
Wound and pain scores for the 4-day follow up study group B (*N* = 200). (**a**) The proportion of dogs with a wound score > 1, and (**b**) the proportion of dogs with a pain score > 0, with 95% confidence intervals

Wounds scores were ‘0–1’ in 89.5% of dogs on the first day post-op, and ‘2’ in 10.5% of dogs. By day two, the number of dogs with a score of ‘2’ had decreased to 7.1%. The pain score was ‘1’ in 20.5% of dogs, and ‘2’ in 0.5% on the day following surgery. No dogs showed a pain score of ‘2’ or more by the second day post-op, and 8.1% showed a score of ‘1’ (mild discomfort). The results are summarised over the four days in Fig. 1, indicating the proportion of dogs with of wound scores greater than 1, and pain scores greater than 0.

The statistical model examined the study group (group A or group B), age, weight, and surgery-time:sex interaction, against the three classifications of complications as an outcome. There was no evidence of an association between the number of major, minor, surgery site specific, or total complications seen, and the study group, age, sex or weight. There was an association between all complications and longer surgery time (effect size = 1.01, *p* = 0.02), as well as major complications and longer surgery time in males (effect size 1.03, *p* = 0.03).

## Discussion

**Fig. 2:**
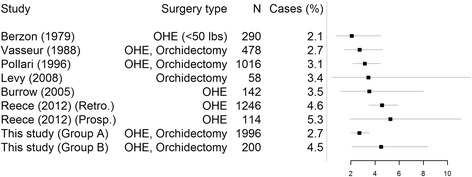
Comparative plot of reported incidence of surgical site specific infection and/or complications. Data shown as percentage of cases with 95% confidence intervals

This study demonstrated that dogs can be sterilised with a low level of surgical complications in a low-income setting without the use of routine post-operative antibiotics. The incidence of post-operative surgical site complications was similar to that seen in teaching hospitals in developed countries (Fig. 2), as well as to field high-volume sterilisation campaigns [9, 19]. Nevertheless variation will exist in the definition of complications between the studies, so direct comparisons should be interpreted with caution. Ovariohysterectomy and castration are classified as clean surgeries, and previous studies indicate a wound infection rate of 0.0–4.9% in clean surgeries and 4.5–5.9% in clean-contaminated surgery [20–25]. In private practice, Pollari and Bonnett, (1996) [26] saw that major surgical site complications ranged from 1 to 4% for dogs and cats undergoing ovariohysterectomy, castration and onychectomy, but this varied widely between surgeons and between practices. Unfortunately the follow-up period in this study was too short to specifically investigate the development of infection which could take weeks to manifest. The total complication rates seen in this study were nevertheless comparable to other studies [19, 27, 28].

In this population with a high proportion of feral dogs, long term follow is not possible without extensive cost and housing facilities. Additionally, hospitalisation may be a marked change from their natural environment, limiting their movement, possibly decreasing territorial behaviours, providing differing nutrition, and added stress from close human and animal contact. Therefore, long periods of hospitalisation both for research and as standard practice, were ethically difficult to justify for this population. Keeping healthy dogs longer than needed is an additional drain on clinic resources, limiting capacity, and is often not logistically feasible in high volume clinics. Totton et al., (2011) [29] suggested that the close contact of animals in kennels may aid in the spread of infectious diseases, including ectoparasites. Moreover, Eugster et al., (2004) [25] found an association between a longer hospitalisation period and increased surgical wound infection rates. Minimising the time spent in the hospital is therefore likely to be beneficial for the free-roaming dogs, as the added stress of confinement may further negatively impact wound healing [30].

In a study by Burrow, Batchelor and Cripps, (2005) [28], major post-surgery complications, such as haemorrhage, were usually seen within 24 h after surgery, and Pearson (1973) [31] found haemorrhage to be the most common cause of death following an ovariohysterectomy. Our study did not see an elevated proportion of major complications in the 4-day follow up compared to the 24 h follow up. Although there was a higher level of minor complications, and those related to the surgical site, this difference was not statistically supported (*p* = 0.18 and *p* = 0.10). This similarity of complication rates between groups shows some evidence that short follow-up times may not be detrimental to the welfare of free-roaming or feral dogs, provided surgical and sterile technique is of a high standard. It is possible that animals released from the clinic early develop post-surgical complications that go undocumented, and could account for the higher complication rate in the dogs studied for a longer period of time. The WVS ITC sterilises owned dogs daily under the same standards as the free-roaming dogs and internal reports (unpublished) indicate a low incidence of complications in owned animals although a formal comparison with free-roaming animals was beyond the scope of this study.

Under-reporting is an inherent bias in this type of study, especially for minor or incidental complications, and record keeping differs between individuals. Additional bias may have been introduced in the selection of the prospective study participants as randomisation was not possible for logistical reasons. This is no reason to believe that patients included in the prospective cohort were different to the retrospective cohort, as origin populations and surgical protocols were similar. However, a difference in monitoring and record keeping of the prospective cohort was plausible. Veterinarians and students were not directly responsible for the research aspects of the study, only clinical duties, and did not have any incentive to report or handle these cases beyond standard care. Though it is possible the veterinarians were influenced by the study in their clinical decision making and reporting, even though standard protocols exist.

Longer anaesthetic and surgery times, were previously associated with an increase in postoperative surgical site infections [21–25, 28]. This study showed no evidence of a relationship between surgery time and surgical site complications, when taking study type, age, weight and sex into account. This may be due to the effectiveness of short-acting, pre-operative antibiotic use in all animals, or the short follow up time. The use of antibiotics before surgery was justified because student surgeries were often expected to take longer than 90 min and Vasseur et al., (1988) [21] saw a 1.6 times increase in infection in student surgeries without prophylactic antibiotic use. Burke, (1961, 1973) [32, 33] showed that antimicrobials given roughly one hour before surgery to maximise the MIC in the blood during the procedure had a favourable effect on wound infection outcomes. However, post-operative administration did not influence wound outcomes in the same study.

A particularly controversial area of surgical sterilisation programmes relates to the use of antimicrobials. Whilst it may seem intuitively sensible to administer a longer course of antibiotics to dogs sterilised in low income settings where ensuring high levels of sterility can be challenging, there are increasing concerns over antimicrobial resistance linked to inappropriate and excessive use of antibiotics. The British Small Animal Veterinary Association (BSAVA), and the Association of Shelter Veterinarians in the USA, advise against antimicrobial use in castrations and ovariohysterectomy, unless the surgery is expected to be prolonged or contaminated [20, 21, 34, 35]. In contrast, the current guidelines for animal birth control (ABC) programmes in India advise 3–5 days of broad-spectrum postoperative antibiotics [15]. Antimicrobial use has been increasing in recent years in India, and isolated studies have shown high levels of resistance in human health care settings [36]. This has stimulated a review of policies within the Indian government on antimicrobial use, including within the veterinary sector, for which the controls are currently limited. Unnecessary use of antimicrobials have other disadvantages including increasing the cost of neutering programmes, causing host microbiome disruption, and drug reactions [37]. In light of these disadvantages, the minimal use of antimicrobials would therefore be beneficial, but there is a paucity of studies which have assessed whether the guidelines from developed countries can be safely applied to low resource sterilisation clinics. In the UK setting, antimicrobials have been administered post-operatively to compensate for poor aseptic technique during surgery [38, 39]. This may also provide the foundation for their use post-operatively in low resource settings. However, this study maintained high aseptic standards under these conditions, precluding the need for additional post-operative antimicrobials. Limiting unnecessary antimicrobial use in these settings reduces costs, could improve compliance to recommended usage, and follows responsible use guidelines to prevent antimicrobial resistance.

In this study, a wound scoring system was used as an objective measure of wound appearance to gauge whether animals were ready for release. The majority of animals, had evidence of acceptable wound healing within 2 days post-surgery. Cases showed a higher proportion of wounds scores > 1 and pain scores > 0, but a higher score did not necessarily imply that an intervention was needed. The scoring may be a helpful tool in assessing whether an animal should be released or requires monitoring, and needs further validation. In this setting, the wound scoring does provide a documented check of each individual animal, necessary when a large volume of patients are seen by a team of veterinarians, ensuring good practice in follow-up care.

This study showed low postoperative pain scores in the majority of animals, with no animals showing severe pain. Poor pain management can contribute to delays in wound healing [40] or result in self-inflicted trauma which also delays healing [14]. The generally accepted multimodal, pre-emptive analgesic approach [41] used in this study may contribute to the good post-operative recovery seen and is an essential factor to consider in sterilisation clinic protocols.

## Conclusions

This study demonstrated that high volume, low-resource sterilisation of dogs can be performed with a low incidence of surgical complications and low mortality. Importantly, we have shown that this can be achieved without the administration of postoperative antibiotics, provided high aseptic and surgical standards are followed and individual assessment takes place for antimicrobial and monitoring requirements with longer hospitalisation as needed, without compromising the welfare of the patients.

## Abbreviations.

WVS: Worldwide Veterinary Service
ITC: International Training Centre
ABC: Animal Birth Control
OHE: ovariohysterectomy.
